# Video image-based analysis of single human induced pluripotent stem cell derived cardiomyocyte beating dynamics using digital image correlation

**DOI:** 10.1186/1475-925X-13-39

**Published:** 2014-04-07

**Authors:** Antti Ahola, Anna L Kiviaho, Kim Larsson, Markus Honkanen, Katriina Aalto-Setälä, Jari Hyttinen

**Affiliations:** 1 Computional Biophysics and Imaging Group, Department of Electronics and Communications Engineering, and BioMediTech, Tampere University of Technology, Tampere, Finland; 2 Heart Group, BioMediTech, University of Tampere, Tampere, Finland; 3 Pixact Oy, Postitorvenkatu 16, FI-33840 Tampere, Finland; 4 Heart Hospital, Tampere University Hospital, Tampere, Finland; 5 Medical School, University of Tampere, Tampere, Finland

**Keywords:** Cardiomyocyte mechanic functionality, Velocity vector analysis, Minimum quadratic difference method

## Abstract

**Background:**

The functionality of a cardiomyocyte is primarily measured by analyzing the electrophysiological properties of the cell. The analysis of the beating behavior of single cardiomyocytes, especially ones derived from stem cells, is challenging but well warranted. In this study, a video-based method that is non-invasive and label-free is introduced and applied for the study of single human cardiomyocytes derived from induced pluripotent stem cells.

**Methods:**

The beating of dissociated stem cell-derived cardiomyocytes was visualized with a microscope and the motion was video-recorded. Minimum quadratic difference, a digital image correlation method, was used for beating analysis with geometrical sectorial cell division and radial/tangential directions. The time series of the temporal displacement vector fields of a single cardiomyocyte was computed from video data. The vector field data was processed to obtain cell-specific, contraction-relaxation dynamics signals. Simulated cardiomyocyte beating was used as a reference and the current clamp of real cardiomyocytes was used to analyze the electrical functionality of the beating cardiomyocytes.

**Results:**

Our results demonstrate that our sectorized image correlation method is capable of extracting single cell beating characteristics from the video data of induced pluripotent stem cell-derived cardiomyocytes that have no clear movement axis, and that the method can accurately identify beating phases and time parameters.

**Conclusion:**

Our video analysis of the beating motion of single human cardiomyocytes provides a robust, non-invasive and label-free method to analyze the mechanobiological functionality of cardiomyocytes derived from induced pluripotent stem cells. Thus, our method has potential for the high-throughput analysis of cardiomyocyte functions.

## Introduction

The withdrawal of drugs already on the market is most commonly due to cardiac side effects. Cardiac safety analyses are currently done using animals as model organisms and/or ectopic expression of single ion channels in non-cardiac human cells [1]. These applications do not provide an optimal platform to explore the conditions in human cardiac cells. Unfortunately, human cardiomyocytes (CMs) have been very challenging to study, since the myocardial biopsy is a high-risk procedure and primary CMs dedifferentiate quickly and stop beating in cell culture conditions. Also, the available methods to measure the functionality of a cardiomyocyte (CM) are challenging and do not provide high throughput.

Moreover, recent developments in stem cell technology, namely the invention of induced pluripotent stem (iPS) cells, have increased the need for new methods to characterize cells derived from iPS cells. iPS cells can be obtained from any individual by reprogramming already differentiated mature cells such as skin fibroblasts into a pluripotent state [2]. Therefore, by using iPS cells it is possible to obtain genetically defined human pluripotent cells that can be differentiated into the cell type of interest, for example CMs [3]. Recently, it has been shown that human iPS cell-derived CMs have proper electrophysiological properties and assays using these cells can provide a reliable alternative to preclinical in vitro testing [4].

The functional measurement of single CMs has traditionally been laborious and time consuming. There are a few tools available for the study of the electrical properties of individual cells. Patch clamp is a commonly used method for analyzing the functionality of single CMs, but this technique requires special, relatively expensive instrumentation, and laborious manual work that requires highly skilled personnel [5]. Microelectrode arrays (MEA) provide a platform for the analysis of larger aggregates of cells with less manual work. Due to the dimensions of the electrodes and the distances between the electrodes, however, they are not suited for single cell functionality studies [6]. Voltage sensitive dyes such as di-8-ANEPPS provide one solution for the analysis of single CMs. This method, however, is based on fluorescence imaging and the dyes interact with some ion channels, e.g. the product of the human Ether-à-go-go-Related Gene (hERG), and thus potentially alter the electrophysiological properties of the cells [7].

The electrical functionality of single CMs does not directly reveal the mechanical properties of the cells. Atomic force microscopy (AFM) can be used to quantify the mechanical properties of CMs, e.g. force. However, AFM is not well suited for long-term measurements because it interferes with the cell [8]. Cellular electric impedance measured with well plate integrated electrodes is also used to measure the beating characteristics of cardiomyocytes [9]. The spatial resolution of the method is, however, not high enough to study the movements within the cell in detail. High-speed video microscopy can be used to obtain information from the beating cycle. Such methods quantify the movement of single CMs with no intervention. By analyzing the movements of the cells, it is possible to receive data from the mechanobiological functionality of the cell and to combine the data with electrical measurements to understand electro-mechanical coupling. Traditional video-based CM analysis methods [10,11] may not, however, be optimal for the study of single iPS cell-derived CMs. The sarcomere structure of iPS cell-derived CMs is not fully organized [12] and, therefore, their beating is less uniform with no main contraction direction. Thus, better methods are required that are robust in the detection of movement signals from these types of cells.

Here, we propose a robust, non-invasive method for the analysis of the beating dynamic of single CMs with no clear axis of contraction by using recorded microscope videos. The method is based on digital image correlation (DIC), more specifically its subtype the minimum quadratic difference (MQD) method that has been developed mainly for particle image velocimetry (PIV). Further, we use sectorial derivation of movement directions. The aim is to provide detailed physical information on the dynamics and timing of the contraction and relaxation of stem cell-derived CMs. The previous methods used to analyze video data [10,11] are not well suited for the analysis of heterogeneous beating. Our method is specifically aimed towards the use of CMs derived from iPS cells. We present validation tests of the method using artificial displacement images and current clamp recordings from human iPS cell-derived CMs. Since the CMs derived from the cell line used here have not previously been fully characterized, we also briefly provide the biological characterization data.

## Materials and methods

### Ethics statement

The study was approved by the Ethical Committee of the Pirkanmaa Hospital District (R08070). A written informed consent from participants has been obtained.

### Cell culture

Primary fibroblasts were obtained from skin biopsy and cultured under fibroblast culturing conditions: Dulbecco’s modified eagle medium (DMEM, Lonza, Switzerland) containing 10% FBS, 2 mmol/l L-glutamine and 50 U/ml penicillin/streptomycin. 293FT-cells (Invitrogen, CA, USA) were maintained similarly with 1% non-essential amino acids (NEAA, Cambrex, NJ, USA). Plat-E-cells (Cell Biolabs, CA, USA) and irradiated or mitomysin C (Sigma-Aldrich, MO, USA) treated mouse embryonic fibroblast (MEF, Millipore, MA, USA) cells were cultured in the same conditions but without antibiotics. iPS cells were cultured with MEF cells as feeders in KSR-medium: knockout (KO)-DMEM (Invitrogen) containing 20% KO-serum replacement (KO-SR, Invitrogen), NEAA, L-glutamine, penicillin/streptomycin, 0.1 mmol/L 2-mercaptoethanol, and 4 ng/ml basic fibroblast growth factor (bFGF, R & D Systems Inc., MN, USA).

### iPS cells

iPS cell lines were established from the dermal fibroblasts of a 55 year old female using lentivirus infection followed by retrovirus infection into the fibroblasts. The following cells, plasmids and reagents were used: 293FT-cells, Plat-E-cells, pLenti6/UbC/mSlc7a1-vector (Addgene, MA, USA), ViraPower™ Packaging Mix (Invitrogen), Lipofectamine™ 2000 (Invitrogen), pMX retroviral vector (hOCT3/4, hSOX2, hKLF4 or hc-MYC, Addgene), and Fugene 6 (Roche Diagnostics, Germany). The full and detailed protocol has been described earlier [2,13]. Two iPS cell lines from the same individual were used for the studies: UTA.04602.WT and UTA.04607.WT.

### Characterization of iPS cells

**Reverse transcription polymerase chain reaction (RT-PCR).** Total RNA was collected from the iPS cells at passage 6 and purified with a NucleoSpin RNA II -kit (Macherey-Nagel, Germany). cDNA conversion was carried out with a high-capacity cDNA RT -kit (Applied Biosystems, CA, USA) using 200 ng of RNA. RT-PCRs were carried out with Dynazyme II (Finnzymes Oy, Finland) using 1 μl of cDNA as a template and 5 μM primers. As positive controls for exogenous primers, PCR was also carried out using the transfected plasmids (hOCT3/4, hSOX2, hKLF4, and hc-MYC) as templates. Primers and reaction conditions for iPS cell characterization [2] and PCR-primers for different germ layer markers [13] have been described earlier. β-actin and glyceraldehyde 3-phosphate dehydrogenase (GAPDH) were used as housekeeping control genes. **Immunocytochemistry for pluripotency.** iPS cells at passage 8 were fixed with 4% paraformaldehyde (PFA, Sigma-Aldrich) and stained with anti-Oct3/4 (1:400, R & D Systems), anti-TRA1-60 (1:200, Millipore), anti-Sox2, anti-Nanog, anti-SSEA4, and anti-TRA1-81 (all 1:200, Santa Cruz Biotechnology, CA, USA). The secondary antibodies (1:800, Invitrogen) were Alexa-Fluor-568-donkey-anti-goat-IgG, Alexa-Fluor-568-goat-anti-mouse-IgM, or Alexa-Fluor-568-donkey-anti-mouse-IgG. Vectashield mounting medium with DAPI (4′,6-diamidino-2-phenylindole, Vector Laboratories Inc., CA, USA) was used to stain nuclei. **Karyotype analysis.** A commercial company (Medix laboratories, Finland) defined the karyotypes of the iPS cell lines by using G-banding chromosome analysis according to standard protocol. **Formation of embryoid bodies (EBs).** EBs were cultured without feeder cells in EB-medium (KO-DMEM with 20% fetal bovine serum (FBS), NEAA, L-glutamine and penicillin/streptomycin) without bFGF for 5 weeks. RNA isolation and reverse transcription from the EBs was performed as described above. The expression of markers characteristic of ectoderm, endoderm, and mesoderm development in EBs was determined using RT-PCR (see above).

### Cardiac differentiation and characterization

CM differentiation was performed by co-culturing iPS cells together with END-2-cells. END-2-cells were cultured as described earlier [14]. To initiate CM differentiation, undifferentiated iPS cell colonies were dissected mechanically into aggregates containing a few hundred cells and placed on the top of Mitomycin C -treated END-2 cells in KSR-culture medium without fetal bovine serum, serum replacement, or basic fibroblastic growth factor. Ascorbic acid (Sigma-Aldrich) was also added into the medium with a final concentration of 2.92 mg/ml [15]. The differentiating cell colonies were monitored by microscopy daily and the medium was changed after 5, 8, and 12 days of culturing. After 14 days, the 10% SR was added to the medium and ascorbic acid was no longer used. **RT-PCR for cardiac markers.** RNA was collected from beating cardiac cells and transcribed into cDNA as described for the pluripotent cells above. The reverse transcription polymerase chain reaction (RT-PCRs) were also carried out in a way similar to that of the pluripotency markers and primers of cardiac markers that have been described earlier [13]. **Immunocytochemical staining.** The spontaneously beating areas of the colonies were mechanically excised and treated with collagenase A (Roche Diagnostics) as described by Mummery et al. [14]. Seven days after dissociation, the cells were fixed with 4% paraformaldehyde for immunostaining with anti-cardiac-troponin-T (1:1500, Abcam, MA, USA), anti-α-actinin (1:1500, Sigma-Aldrich), anti-myosin-heavy-chain (MHC, 1:100, Millipore), anti-atrial-myosin-light-chain (MLC2a, 1:300, Abcam), and anti-ventricular-myosin-light-chain (MLC2v, 1:150, Abcam). The secondary antibodies (1:800, Invitrogen) were Alexa-Fluor-568-donkey-anti-goat-IgG, Alexa-Fluor-568-coat-anti-mouse-IgG, Alexa-Fluor-488-donkey-anti-rabbit, and Alexa-Fluor-488-donkey-anti-mouse. Vectashield mounting medium with DAPI was used to stain the nuclei. Dissociated CMs were prepared for video recording in the same way as for immunocytochemical staining.

### Video microscopy

Videos of the dissociated spontaneously beating single CMs were recorded using video microscopy. Both iPS cell lines were used in the recordings and they gave identical results. Thirteen CMs were video-recorded for 30 s at 30 frames per second under sterile conditions. The CMs were visualized using a Nikon Eclipse TS100 (Nikon Corporation, Japan) microscope and monochrome 8 bit videos were acquired with an Optika DIGI-12 (Optika Microscopes, Italy) camera mounted on the microscope. Additionally, two CMs were videoed and their concurrent action potentials were acquired with current clamp measurement for combined functionality verification. In this series, a high resolution 14 bit Andor XION 885 (Andor Technology, UK) camera mounted on an Olympus IX51 (Olympus Corporation, Japan) microscope was used. Transmission images were acquired for 60 s at 50 frames per second using TILLvisION (TILL photonics GmbH, Germany).

### Digital image correlation analysis

The term DIC refers to methods that acquire images and perform analysis for full-field shape, deformation and/or motion measurements [16]. The images are divided into small sub-regions where the grayscale values are cross-correlated between the consecutive image frames to provide a displacement map that indicates the movements of the scene [16]. Standard cross-correlation analysis emphasizes bright pixels due to the multiplication of intensity values [16]. In CM images, however, all image pixels regardless of their grayscale value can contribute to the motion analysis. Therefore, the weighting of bright pixels in the standard cross-correlation analysis is a clear disadvantage. In this study, we removed this disadvantage by using the MQD method [17] that puts equal weight to all image pixels. Additionally, MQD has been shown to be more accurate than other PIV evaluation methods based on correlation [18]. The MQD method was originally developed to evaluate PIV recordings. It uses a least-square principle to obtain the velocity vector field across the image based on two consecutive video frames [16]. Image sub-regions (i, j) are compared between the consecutive image frames (I_1_ and I_2_) using the function (1):

(1) $$S_{i,j}\left(\mathrm{dx},\mathrm{dy}\right)=\sum_{x=-N/2}^{N/2}\overset{N/2}{\underset{y=-N/2}{\sum }}{\left[I_{1}\left(i+x,j+y\right)-I_{2}\left(i+x+\mathrm{dx},j+y+\mathrm{dy}\right)\right]}^{2},$$

where x and y are the indices to the pixels inside the sub-region of size [N, N]. The sub-region in the second frame I_2_ is shifted in x- and y-directions by dx, dy to obtain a value at point (dx, dy) in the quadratic difference map S_i,j_ of sub-region (i, j). The computational ranges of dx and dy can be freely selected to match the application. The location of the minimum value in S_i,j_ reveals the medial displacement of the scene inside the sub-region (i, j). Due to small displacements between CM frames, the necessary sub-pixel accuracy in displacement estimation is obtained by sub-pixel fitting on the minimum value (dx, dy) in S_i,j_ with a 1-dimensional 3-point Gaussian interpolation fitting function (2) to determine the correlation peak [19].

$$\text{Δ}x=\frac{\ln\left(S\left(\mathrm{dx}-1,\mathrm{dy}\right)\right)-\ln\left(S\left(\mathrm{dx}+1,\mathrm{dy}\right)\right)}{2\left[\ln\left(S\left(\mathrm{dx}+1,\mathrm{dy}\right)\right)-2\ln\left(S\left(\mathrm{dx},\mathrm{dy}\right)\right)+\ln\left(S\left(\mathrm{dx}-1,\mathrm{dy}\right)\right)\right]},$$

(2) $$\text{Δ}y=\frac{\ln\left(S\left(\mathrm{dx},\mathrm{dy}-1\right)\right)-\ln\left(S\left(\mathrm{dx},\mathrm{dy}+1\right)\right)}{2\left[\ln\left(S\left(\mathrm{dx},\mathrm{dy}+1\right)\right)-2\ln\left(S\left(\mathrm{dx},\mathrm{dy}\right)\right)+\ln\left(S\left(\mathrm{dx},\mathrm{dy}-1\right)\right)\right]}$$

### Cardiomyocyte analysis with MQD

**Figure 1:**
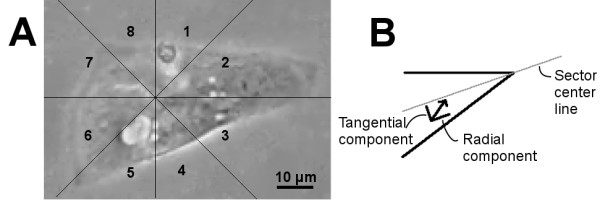
**Beating analysis framework. A**: The cell is divided into 8 sectors, each being of 45°, with the center point being at the observed beating focus point of the cell. The sectors are numbered in a clockwise manner. **B**: The radial and tangential components of the velocity vectors in each sector are calculated with regard to the sector centerline going through the beating focus point.

Single CMs recorded on video were manually segmented for MQD analysis. The non-moving parts of the cell were cropped outside of the region of interest to decrease processing time and noise. To obtain a cell-specific coordinate system for the beating analysis, the beating focus point of the cell is selected by visual approximation from the video and the region of interest is divided into 8 sectors, each comprising a 45-degree sector from the beating focus (Figure 1A). This enables the analysis of the inconsistent beating patterns of iPS-derived CMs. For each velocity vector in a sector, two dot products are calculated. First with regard to the center line of the sector, to calculate the approximate radial component, and second with regard to the normal of the center line, to calculate the approximate tangential component (Figure 1B). The centerline normals pointing towards sectors 1–4 were selected for sectors 1–4, and the normals pointing towards 5–8 for sectors 5–8. For each sector, the sum of these vector components was calculated. In total, 16 different signals, 8 radial and 8 tangential signals, were obtained from a video.

The analysis was conducted using open source Matlab algorithm mpiv [20]. A 16×16 px subwindow size with a 0.5 overlap ratio was used. The resulting vectors were smoothed using a median filter. Possible stray vectors were determined and removed if the vector was outside the range of 2.5 times the standard deviation from the mean value. Kriging interpolation was used to assign values for vectors that did not have applicable values. Finally, weighting was used to smooth the vector field using a 3 × 3 kernel $\begin{array}{ccc} 1 & 2 & 1\\ 2 & 4 & 2\\ 1 & 2 & 1 \end{array}$ as a low-pass filter.

### Data verification

**Figure 2:**
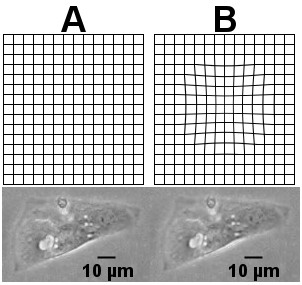
**Artificial data set created from a cardiomyocyte image.** An even grid and a cardiomyocyte image are shown to illustrate the effect of the artificial deformation that was used to create the data set. **A**: An even grid and a cardiomyocyte image without the artificial deformation. **B**: An even grid and a cardiomyocyte image with the described deformation applied, γ = 10.

The proposed beating analysis was verified using artificial displacement images. We modified still CM images so that they modeled the displacement of the pixels during CM beating with known displacement. An image distortion filter [21] was modified and used on a CM image to create artificial distortions that resembled the various stages of a beating iPS cell-derived CM with no main contraction axis. The resulting images were analyzed using the MQD method. Figure 2 illustrates the effect of the artificial distortion on an even grid image and on a CM image.

The artificial images for the video were constructed by stretching the cell with the distortion γ. Each point (x, y) in the original image within a set radius from the determined beating focus was mapped onto a virtual half-sphere of radius R, and a new distance X to the beating focus point was set-based on the desired distortion factor γ, as done in the original image distortion filter.

With this method, an image of a cell was modified with varying values of γ and combined to a video to get artificial cell data resembling that of a beating cell. Artificial images were created using 5 different γ values: -1, -2, -4, -7, and -10. The video was created from a total of 51 frames representing two beats that comprised 10 still frames, 5 frames with decreasing γ values, 5 frames with increasing γ values, 11 still frames, 5 frames with decreasing γ values, 5 frames with increasing γ values, and finally 10 still frames. Figure 2A shows an unmodified, original image of the cell and Figure 2B an image distorted using the explained method with γ = -10. The values of X define the displacement that can be compared with the results of the MQD analysis due to symmetry.

### Noise resistance testing

The noise resistance of the proposed method was tested by adding multiplicative speckle noise to each frame of the generated artificial video data that was obtained from modifying a CM image, as explained above. The cell size was 6796 pixels. Speckle noise was added to each image using the equation $J=I+\sqrt{12*V}*I*I_{r}$, where I is the original image, J is the resulting image, V is variance and I_r_ is uniformly distributed random noise between values -0.5 and 0.5, with mean of 0. 8. The following noise variances were used: 0, 0.03, 0.05, 0.07, 0.09, 0.011, 0.013, and 0.015.

### Beating analysis of cardiomyocytes

The analysis was carried out for the 13 CMs recorded on video. The time required for each phase of beating was measured: the contraction, the time it stayed contracted, the relaxation time, and the time it stayed relaxed. The beating frequency was also measured.

### Current clamp measurement

To further verify the findings, the proposed video analysis was conducted from video data recorded from iPS cell-derived CMs with concurrent current clamp measurement. Action potentials were recorded using the Axopatch 200B patch clamp amplifier connected to an acquisition computer via AD/DA Digidata 1440 (Molecular devices, USA). The measurement was carried out at room temperature in gap free mode using the standard current clamp configuration in perforated patch mode. The HEPES (4-(2-hydroxyethyl)-1-piperazineethanesulfonic acid)-based extracellular perfusate for current clamp recordings comprised (in mmol/l): 143 NaCl, 5 KCl, 1.8 CaCl_2_, 1.2 MgCl_2_, 5 glucose, and 10 HEPES. The pH was adjusted to 7.4 with NaOH and the osmolarity set to 300 ± 2 mOsm (Gonotec, Osmomat 030, Labo Line Oy, Finland). The intracellular solution comprised (in mmol/l): 122 KMeSO_4_, 30 KCl, 1 MgCl_2_, and 10 HEPES. KOH was used to set pH to 7.15 and the osmolarity was set to 295 ± 2 mOsm. Amphotericin B (Sigma-Aldrich) was used as a membrane perforation agent and was dissolved in dimethyl sulfoxide to a final concentration in the patch pipette of 0.24 mg/ml. Current-clamp recordings were digitally sampled at 20 kHz and filtered at 5 kHz using the lowpass Bessel filter on the recording amplifier.

### Combining current clamp and video analysis

TTL synchronization pulses (1 pulse per frame) were delivered by the digital signal processor-driven imaging control unit (programmed in TILLvisION) to synchronize the transmission frames and current clamp data sampling. The pulses and current clamp data were concurrently sampled using 2 channels in current clamp. The video data obtained simultaneously with the current clamp measurement was processed using the proposed method.

## Results

### Characterization of induced pluripotent stem cells

**Figure 3:**
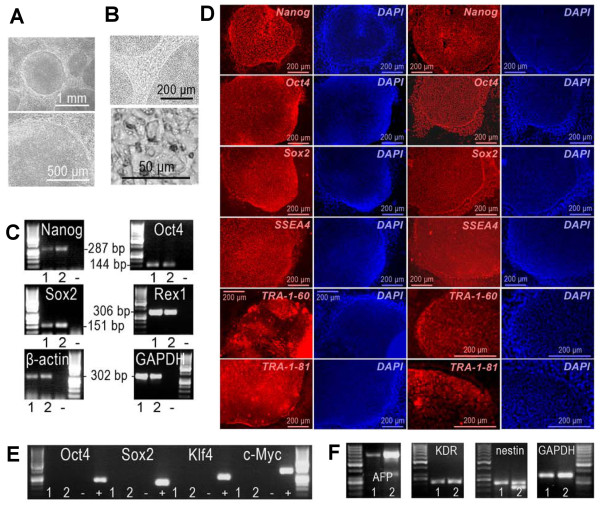
**Characterization of iPS cells for their pluripotency.** Number 1 represents the cell line UTA.04602.WT and 2 the line UTA.04607.WT. **A**: iPS cells formed typical colonies for pluripotent stem cells that are rather compact and round in shape. **B**: The iPS cell colonies typically had well defined edges and distinct cell borders, and the iPS cells had a high nucleus to cytoplasm -ratio and a large nucleoli characteristic for stem cells. **C**: Endogenous pluripotency gene expression was studied using RT-PCR. Nanog, Oct4, Sox2 and Rex1 were all expressed at mRNA level in the iPS cells. β-actin and GAPDH were used as housekeeping control genes for both endogenous and exogenous markers. **D**: The expression of pluripotency genes was also studied at the protein level by immunocytochemical staining. The iPS cell expressed several markers for the pluripotent state: Nanog, Oct4, Sox2, SSEA4, TRA-1-60, and TRA-1-81 (all in red). Pictures in the left panel are from the line UTA.04602.WT and the ones on the right side are from UTA.04607.WT. Blue in all pictures indicates the DAPI staining of nuclei. **E**: Using RT-PCR, it was shown that all the transgenes were silenced in the iPS cells. Negative control is marked with “-” and positive control with “+”. **F**: Embryoid body (EB) -assay was used to define the pluripotency of the iPS cells *in vitro*. Markers for all three germ layers were detected from the EBs formed from both cell lines. Alpha*-*fetoprotein (AFP) was used as a marker for endoderm, kinase insert domain receptor (KDR, also known as vascular endothelial growth factor receptor 2 (VEGFR-2) was used as a marker for mesoderm and nestin was used as an ectoderm marker. GAPDH was used as an endogenous control gene.

**Figure 4:**
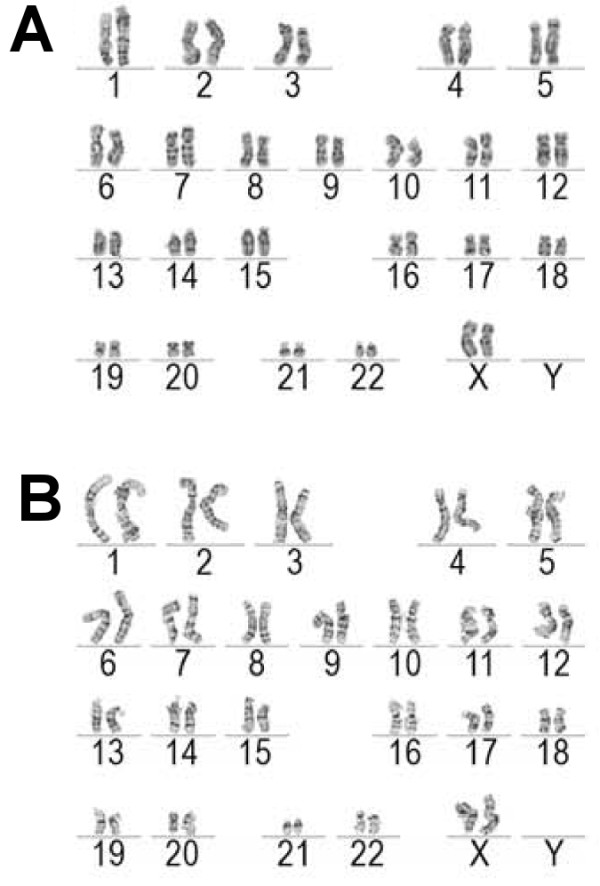
**Karyotype analysis from iPS cells. A** &**B**: The lines were verified for normal karyotypes (G: UTA.04602.WT and H: UTA.04607.WT).

The generated iPS cells were first characterized by the morphology of the cell colonies and the individual cells that exhibited similar characteristics to those of human embryonic stem cells: compact and round in shape (Figure 3A), defined edges and distinct cell borders (Figure 3B). The iPS cells also expressed endogenous pluripotent marker genes at the mRNA level that was shown by RT-PCR (Figure 3C). The expression of pluripotency genes also at the protein level was demonstrated by the immunocytochemical staining of different markers for pluripotent stem cells (Figure 3D). On the other hand, transgene expression was turned off in the iPS cells (Figure 3E). To confirm the pluripotent state of the iPS cells, an EB formation assay was carried out. The cells from the EBs were shown to express marker genes from all three germ layers: endoderm, ectoderm, and mesoderm (Figure 3F). The generated cell lines were also analyzed for their karyotypes and were both found to be normal (Figure 4A and B).

### Characterization of the cardiomyocytes differentiated from iPS cells

**Figure 5:**
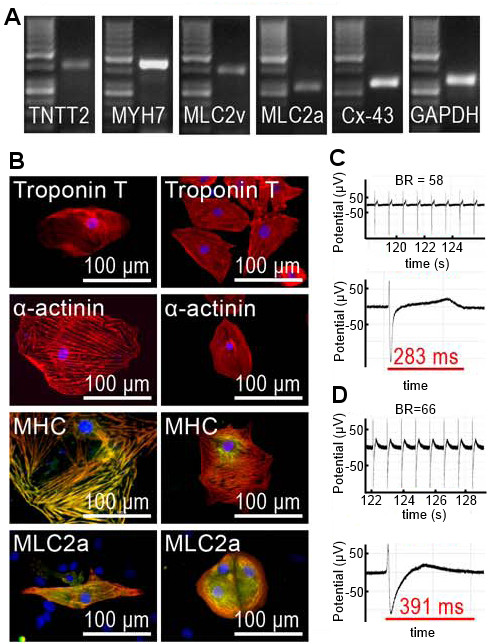
**Cardiomyocytes differentiated from the iPS cells. A**: Several cardiac markers were discovered using RT-PCR indicating their expression at mRNA level. Data from UTA.04602.WT is shown here. **B**: By immunocytochemical staining it was shown that the iPS cell-derived cardiac cells express proteins specific for cardiomyocytes. Cardiac troponin T, α-actinin, myosin heavy chain (MHC) and atrial myosin light chain 2 (MLC2a) were detected from the cells. The pictures on the left side are from the line UTA.04602.WT and the pictures on the right side are from UTA.04607.WT. In the pictures showing MHC and MLC2a with green fluorescent, red indicates troponin T and in all pictures DAPI staining of nuclei is seen in blue. **C** &**D**: A micro electrode array (MEA) was used to define the electrical properties of the iPS cell-derived cardiomyocytes. The beating rates (BR) and field potential durations (FPD) of cell aggregates were evaluated (B: UTA.04602.WT, BR = 58, FPD = 283 ms and C: UTA.04607.WT, BR = 66, FPD = 391 ms).

Generated iPS cells were differentiated into spontaneously beating CMs that expressed different cardiac markers. Using RT-PCR, it was shown that troponin T (TNTT2), MLC2v, MLC2a, connexin-43 (Cx-43), myosin heavy chain (MYH7), hERG, and GATA4 were expressed in the cells (data not shown for hERG and GATA4) (Figure 5A). The expression of cardiac marker genes at the protein level was also confirmed (Figure 5B). With immunocytochemical staining, it was also shown that both atrial and ventricular cells were present among the iPS cell-derived CMs (data not shown). The electrical properties of the iPS cell-derived CMs were also characterized using a microelectrode array that revealed that the cell aggregates exhibited appropriate beating rates and field potentials (Figure 5C and D).

### Beating signals of iPS cell-derived CMs

**Figure 6:**
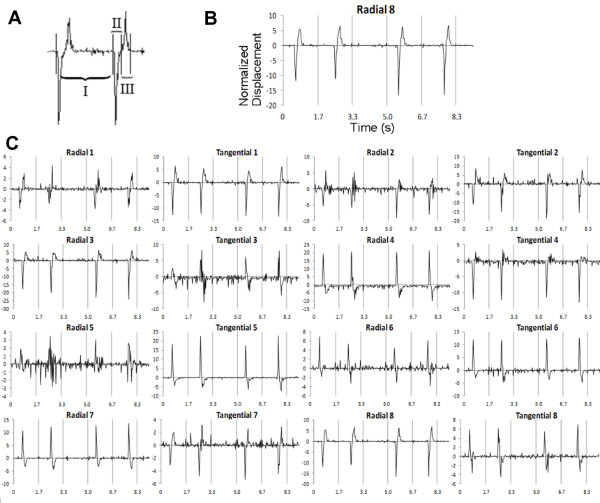
**The beating signals obtained from an iPS cell-derived CM. A**: The classification of beating phases. The time between each individual beat is marked with I. Contraction movement is defined as negative movement (marked with II) and relaxation movement as positive (marked with III). **B**: An enlarged image of the sector 8 radial component. **C**: The beating signals of each sector obtained from one of the measured CMs. For various sectors, the contraction seems to push certain areas away from the perceived beating focus point. This results in an upward peak in lieu of a downward peak due to the fusiform nature of the beating. While most sectors produce a signal of high quality, sectors such as radial 5 have too little movement to generate a well-formed displacement signal.

The method was applied on iPS-derived CMs to obtain beating signals. One such signal that shows two beats is shown in Figure 6A. For radial components, contraction was defined negative (Figure 6A II) and relaxation positive (Figure 6A III). Figure 6B illustrates a typical signal.

From the 13 measured cells, the time parameters of the different phases of the beating were calculated from the sum displacement signal using the proposed method. The phases were the following: contraction, time the cell was contracted, relaxation, and the time the cell was relaxed. The average values of three analysis signals for each cell were calculated. The results for each cell measurement are presented in Table 1.

**Table 1:** Multiple cell analysis

**Cell**	**Frequency (bpm)**	**Contraction (ms)**	**Contracted (ms)**	**Relaxation (ms)**	**Relaxed (ms)**
1	12.03	451	18	571	3949
2	18.20	419	0	612	2265
3	22.78	321	0	444	1868
4	23.64	360	1	447	1730
5	24.12	164	0	202	2122
6	24.56	427	8	535	1472
7	25.98	179	0	346	1784
8	26.34	225	13	594	1446
9	29.13	225	0	318	1517
10	36.45	267	1	324	1053
11	41.82	216	3	295	921
12	43.13	274	30	361	727
13	66.34	209	7	315	373

Results obtained from multiple cell analysis. The times of the different beating phases in milliseconds and the corresponding beats per minute (bpm) were measured from 13 cells.

Figure 6C shows all 16 velocity signals obtained from one of the measured cells.

### Method verification using artificial displacement images

**Figure 7:**
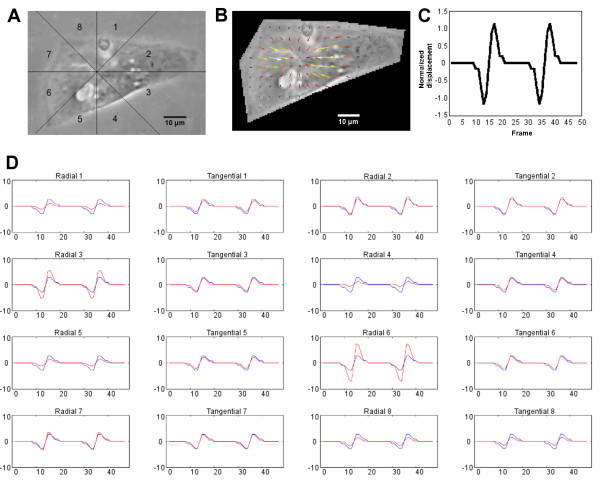
**Comparison of measured and known motion signals in different sectors.** A CM image was modified using image-processing methods with varying parameters to create a video resembling the contraction and relaxation of the cell. In the upper row are illustrated **A**: the sector division, **B**: the velocity vector field during the contraction, and **C**: the known displacement velocity. Since the artificial displacement has circular symmetry, a horizontal cross-sectional displacement is shown. **D**: The proposed method was applied to the video data and the signal marked with red was obtained. A blue signal illustrates the known displacement field.

The DIC analysis results created using artificial displacement images are illustrated in Figure 7. The figure shows (A) the division of the cell into analysis sectors, (B) the displacement vector field during the contraction phase, (C) the known displacement velocity, and (D) the results of the DIC analysis (red) shown with the known displacement velocity (blue). The average correlation coefficient between the known displacement field and the analysis results for all 16 signals was 0.9525.

**Figure 8:**
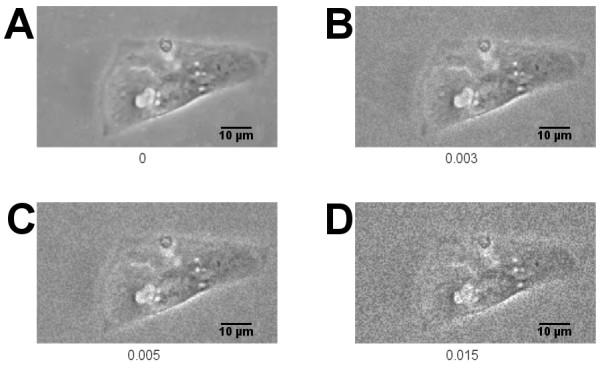
**Assessment of the effect of noise on video beating analysis.** Varying degrees of speckle noise variance were added to a video for testing noise resistance. **A-D**: Example images for four noise variance levels are shown: 0, 0.003, 0.005, and 0.015.

For noise resistance testing, 8 videos with speckle noise were created using different noise variances. The proposed analysis was applied to these videos. For each video, 3 segments were chosen. The average correlation between the resulting signals and the known displacement was calculated as a function of noise variance. The results for three different sectors are shown in Table 2. Example images of different noise levels are shown in Figure 8A-D.

**Table 2:** Analysis result correlation with known displacement

	**Radial component 1**		**Radial component 3**		**Radial component 5**	
**Noise variance**	**AVG**	**SD**	**AVG**	**SD**	**AVG**	**SD**
0	0.964	0.000	0.952	0.000	0.939	0.000
0.003	0.922	0.034	0.956	0.005	0.781	0.150
0.005	0.761	0.111	0.950	0.013	0.532	0.152
0.007	0.480	0.364	0.926	0.076	0.359	0.389
0.009	0.490	0.177	0.894	0.050	0.456	0.209
0.011	0.423	0.342	0.671	0.310	0.334	0.228
0.013	0.183	0.385	0.441	0.250	0.325	0.324
0.015	0.259	0.335	0.435	0.440	-0.041	0.410

The average correlations (AVG) of the analysis results with the known displacement field and their standard deviations (SD). Noise with variances ranging from 0 to 0.015, with example images shown in Figure 6A-D, was added to video frames that comprised the artificial displacement images of a cardiomyocyte. The average correlation of 8 videos between the analysis results and the known displacement was determined, along with the standard deviation.

### Comparison with current clamp recording

Spontaneously beating iPS cell-derived CMs had the characteristics of ventricular- type cells. The cells were beating individually and not part of a larger beating cell cluster.

The velocity vector data was integrated with respect to time in order to obtain position data, and compared with the current clamp data to see the relationship between the mechanical and electrical activity. The time between the peaks of electrical activity and mechanical activity was calculated from the synchronized data.

**Figure 9:**
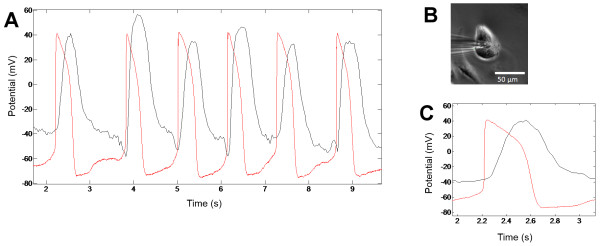
**Combined current clamp and displacement data for a cell. A**: Concurrent action potential and displacement signal, integrated with respect to time from the movement data obtained from the video data, are plotted in the same figure. The action potential data is shown in red and the movement data in black. For current clamp data, the potential in mV is shown, whereas for movement data, the y-axis is arbitrary. **B**: Image from the video data used for the analysis of a CM being recorded with current clamp. **C**: An enlarged part of the signal showing the time difference between the action potential and the displacement signal.

The DIC signal onset occurred after the action potential onset and the action potential declined earlier. Figure 9 shows both signals from one of the recorded cells in the same graph. The basic action potential parameters for the recorded cell are listed in Table 3. The time difference between the peaks of action potentials and the maximum displacement was 306 ms with a 40 ms standard deviation. The result is in line with previously reported values [22].

**Table 3:** Action potential parameters

**Unpatched (bpm)**	**Patched (bpm)**	**Vmax (dV/dT)**	**APD10 (ms)**	**APD50 (ms)**	**APD90 (ms)**	**APA (mV)**
39	42.52	43.08	97.67	279.53	371.00	116.30

CM action potential parameters in current clamp experiment. Beats per minute (bpm) for unpatched and patched cells are listed. Maximal rise velocity (Vmax), action potential durations at 10%, 50% and 90% of repolarization (APD10, APD50, and APD90, respectively), and action potential amplitude (APA) are listed.

## Discussion

We developed a microscope video analysis method to provide accurate and detailed information on the beating motion dynamics of single CMs, especially those derived from stem cells. We observed that our DIC-based methods are promising for the study of the mechanical functions of CMs because they enable the cell geometry-based beating parameters of cells to be calculated. Previously, DIC had been used for the analysis of cell cultures [11] and the morphogenesis of the heart and changes in blood flow during embryogenesis [23]. The displacement vector analysis approach has also been used for the analysis of the motion of living embryos [24]. In these *in vivo* studies, fluorescent particles were injected into the embryos and the motion of the particles inside the heart was analyzed. Our method does not require the invasion of the cell or the use of an artificial tracer and can be used for detailed single cell analysis.

DIC was found to be a viable complement to electrical studies in CM research. In this study, we demonstrated that MQD can be successfully used to analyze single beating CMs. Further, dividing the cell into sectors and calculating the radial and tangential signals for different parts of the cell provides a way to derive basic cell motion directions and thus motion signals related to cell geometry. This enabled the robust detection of all movements. This is especially important for iPS cell-derived CMs that do not beat as uniformly as fully matured native CMs. As also shown here, the beating does not have a main contraction axis and the beating shows fusiform characteristics.

In comparison to other similar methods that analyse contraction [10,11], our analysis that uses a sector approach is advantageous for iPS cell-derived CMs that lack the well-organized structure of mature native cells. Our results show that the eight sectors and directional components provide signals that can be used to analyze the mechanical behavior of an IPS cell-derived CM. Because it does not depend on mathematical estimators such as the principal components of motion vectors, the method is simple and robust. In our method dividing the cell into sectors depends on the visual selection of the beating focus point. While this approach allows flexibility in analysis, it could result in a source of user based error. As the optimal selection of the beating focus point is not inherently clear, automated methods for beating focus point detection well warranted.

Our results indicate that our method provides a reliable signal, and the results of the simulated data show that the method performs well in challenging noise conditions. The sector analysis withstood noise very well and provided high correlation figures compared to simulated movements. In addition, the speckle noise applied in the noise resistance provides a good approximation of video noise. The simulation results indicate that our method is also applicable with relatively low quality video data and that the standard cameras used in microscopes are capable of producing video data that can be used for reliable motion analysis.

We used current clamp, considered as a gold standard for the analysis of single CMs [25] as a reference method to verify the timing of beating. The region of interest for the analysis was partly hampered by the patch pipette that blocked a large part of the view to the cell. Still, the mechanical behavior of the beating cells, which corresponded to electrical activity measured with current clamp, could be detected with the MQD method with good accuracy. Furthermore, we observed that the beating of the cell during the patching was noticeably weaker indicating that patching can alter the function. Thus, non-invasive and non-label methods such as those presented here are well warranted for the detailed functional analysis of CMs.

Some issues should be taken into account when making the video recordings and the motion analysis. For example, the cells in our test were of varying sizes and, despite our best efforts, the video focus was not uniform for all cells. In some videos, noise in the recording and video packing artifacts caused noise in the motion analysis signal. This was especially observed for the cells that had weak beating, videos that were slightly out of focus, and cells that had a uniform surface pattern. The calculated contraction and relaxation times for different cells were, however, similarly proportioned despite the noise. Based on these findings, it can be stated that high quality recordings are not required. Since the method creates the signal based on the moving patterns inside the cell, it can also provide a good signal when the cell is attached to the surface and the outline does not move.

In many cases, the dissociated CMs are attached to the bottom of the culture plate in such a way that the outline of the cell does not move and the movement can only be seen by observing the moving patterns inside the cell. Some cells, however, also had a moving outline. As a result, the cells have varying and fusiform movement patterns. However, our method was able to derive the movement dynamics of all these cells by using the cell geometry-based sectorial and directional summation of the PIV vectors. The sectorial approach has specific benefits for the analysis of cells such as stem cell-derived CMs with varying and inhomogeneous movement patterns inside the cells or with a lack of a main axis of contractility. Due to these reasons, quantifying the extent of the contraction or contractility of the dissociated CMs derived from iPS cells, e.g. in pixels per time unit or movement in micrometers, is not meaningful.

## Conclusions

MQD analysis using a sector approach provides a novel way for the non-invasive and non-labeled function analysis of the biomechanics of CMs, especially those derived from stem cells. Motion analysis in general has clear advantages over other existing methods: it requires very little training for personnel, it does not require external hardware apart from a microscope and a video camera, it can be used for high throughput screening, and it is non-invasive and label-free**.** Motion analysis can also reveal information that is beyond the electrical properties and ion movements of the cardiac cells such as actual biomechanical timing and possible intracellular motion defects. Motion analysis is, therefore, an important addition to any electrical study. Moreover, the motion analysis method may provide an important addition to the traditional way of studying cell functionality, especially the actual mechanical movement, namely the timing of contraction and relaxation of CMs. Our method is especially designed to provide robust motion information from fusiform inhomogeneously beating dissociated CMs derived from stem cells.

In conclusion, these capabilities in conjunction with improved stem cell technologies that produce patient specific cell lines make the proposed system a good candidate for high throughput drug screening, safety analysis, and the basic studies of cardiac diseases using stem cell-derived CMs.

## Abbreviations

AFP: Alpha-fetoprotein; AFM: Atomic force microscopy; APA: Action potential amplitude; APD: Action potential duration; AVG: Average correlation; bFGF: Basic fibroblast growth factor; BR: Beating rate; CM: Cardiomyocyte; Cx-43: Connexin-43; DAPI: 4′,6-diamidino-2-phenylindole; DIC: Digital image correlation; DMEM: Dulbecco’s modified eagle medium; EB: Embryoid body; FBS: Fetal bovine serum; FPD: Field potential duration; GAPDH: β-actin and glyceraldehyde 3-phosphate dehydrogenase; HEPES: 4-(2-hydroxyethyl)-1-piperazineethanesulfonic acid; hERG: Human ether-à-go-go-related gene; iPS: Induced pluripotent stem; KDR: Kinase insert domain receptor; KO: Knockout; KO-SR: Knockout serum replacement; KSR: Knockout serum replacement -medium; MEA: Micro electrode array; MEF: Mouse embryonic fibroblast; MHC: Myosin-heavy-chain; MLC2a: Atrial myosin light chain; MLC2v: Ventricular myosin light chain; MQD: Minimum quadratic difference; MYH7: Myosin heavy chain; NEAA: Non-essential amino acids; PFA: Paraformaldehyde; PIV: Particle image velocimetry; RT-PCR: Reverse transcription polymerase chain reaction; SD: Standard deviation; TNTT2: Troponin T; VEGFR-2: Vascular endothelial growth factor receptor 2.

## Competing interests

A patent application concerning the method has been filed. There are no other conflicts of interests.

## Authors’ contributions

AA developed and tested the method, analyzed the videos, modeled the artificial beating and drafted the manuscript. AK conducted the iPS cell production, CM differentiation and characterization, recorded the videos, and drafted the applicable parts of the manuscript. She also observed the need for the method. KL conducted the patch clamp experiment and drafted the applicable parts of the manuscript. MH participated in conception of the method and assisted with manuscript drafting. KA-S and JH participated in the development of the method, the design and coordination of the study, and helped to draft the manuscript. KA-S also participated in formulating the original necessity for the method. All authors read and approved the final manuscript.
